# Associates of Cardiopulmonary Arrest in the Perihemodialytic Period

**DOI:** 10.1155/2014/961978

**Published:** 2014-11-04

**Authors:** Jennifer E. Flythe, Nien-Chen Li, Shu-Fang Lin, Steven M. Brunelli, Jeffrey Hymes, Eduardo Lacson

**Affiliations:** ^1^Department of Medicine, Renal Division, Brigham and Women's Hospital, Boston, MA, USA; ^2^Department of Medicine, Division of Nephrology and Hypertension, University of North Carolina Kidney Center, UNC School of Medicine, 7024 Burnett-Womack CB No. 7155, Chapel Hill, NC 27599-7155, USA; ^3^Fresenius Medical Care North America, Waltham, MA, USA; ^4^DaVita Clinical Research, Minneapolis, MN, USA

## Abstract

Cardiopulmonary arrest during and proximate to hemodialysis is rare but highly fatal. Studies have examined peridialytic sudden cardiac event risk factors, but no study has considered associates of cardiopulmonary arrests (fatal and nonfatal events including cardiac and respiratory causes). This study was designed to elucidate patient and procedural factors associated with peridialytic cardiopulmonary arrest. Data for this case-control study were taken from the hemodialysis population at Fresenius Medical Care, North America. 924 in-center cardiopulmonary events (cases) and 75,538 controls were identified. Cases and controls were 1 : 5 matched on age, sex, race, and diabetes. Predictors of cardiopulmonary arrest were considered for logistic model inclusion. Missed treatments due to hospitalization, lower body mass, coronary artery disease, heart failure, lower albumin and hemoglobin, lower dialysate potassium, higher serum calcium, greater erythropoietin stimulating agent dose, and normalized protein catabolic rate (J-shaped) were associated with peridialytic cardiopulmonary arrest. Of these, lower albumin, hemoglobin, and body mass index; higher erythropoietin stimulating agent dose; and greater missed sessions had the strongest associations with outcome. Patient health markers and procedural factors are associated with peridialytic cardiopulmonary arrest. In addition to optimizing nutritional status, it may be prudent to limit exposure to low dialysate potassium (<2 K bath) and to use the lowest effective erythropoietin stimulating agent dose.

## 1. Introduction

Hemodialysis (HD) patients experience high rates of cardiovascular morbidity and mortality [1]. Cardiac arrest rates among HD patients exceed those of the general population by 30-fold [1–3]. While the vast majority of such arrests occur in unmonitored settings, arrests during and immediately surrounding the HD procedure (i.e., peridialytic) occur in outpatient HD facilities at a rate of ~7 per 100,000 HD sessions [4]. Postperidialytic arrest outcomes are dismal with 1-year survival rates of 9–15% [1, 5]. Prior analyses have identified low dialysate potassium [4, 6] and calcium [7], extremes of serum potassium [7], and traditional risk factors such as diabetes and underlying burden of cardiovascular disease [4] as risk factors for sudden cardiac arrest (SCA) and sudden cardiac death (SCD) among dialysis patients. Risk factors for a broader class of peridialytic events that include respiratory arrest, nonfatal sudden cardiac events, and cardiopulmonary arrest (CPA) are not well-described. While such events, including extreme hypotension, pulmonary embolus, cerebrovascular disorders, seizures, and respiratory arrests (among others), have lower mortality rates than do SCA events, they are nonetheless significant sources of patient morbidity and stress to ambulatory HD facility staff and patients.

We undertook this study to elucidate patient and HD procedural characteristics that associate with CPA. To capture a wide range of peridialytic events, we defined CPA as the absence of a pulse and/or cessation of spontaneous breathing while in the outpatient dialysis facility. Using data from a large, nationally representative 2010 prevalent, in-center patient cohort, we employed a case-control study design in which we matched patients with in-center CPA (cases) to patients without in-center CPA (controls) on the basis of age, gender, race, and diabetes status. To isolate CPA-associated characteristics from perideath-associated characteristics, we performed additional analyses matching CPA cases to non-CPA controls who survived the study year and, separately, to non-CPA controls who died during the study year.

## 2. Materials and Methods

### 2.1. Study Design and Study Population

We utilized a case-control study design to elucidate patient and procedural risk factors for peridialytic CPA in the ambulatory setting. CPA was defined as absence of apical or carotid pulse (by corollary, blood pressure) and/or cessation of breathing while on HD or immediately before/after the treatment while in the outpatient dialysis facility, as determined by HD center nursing staff at the time of the event. Routine verification of CPA cases demonstrated >99% concordance between the initial CPA attribution and subsequent adjudication based on medical record review by quality reviewers. In-center HD patients from the 2010 prevalent population of Fresenius Medical Care, North America (FMCNA), were eligible for cohort inclusion. CPA cases were identified from the electronic medical record by screening for HD-associated adverse events. Exclusion criteria were age <18 years, traveling or transient patients, and patients with acute kidney injury. A total of 937 patients with CPA (cases) and 76,202 patients without CPA (eligible controls) were identified from 653 FMCNA facilities where at least one CPA event occurred.

### 2.2. Data Collection

All study data were obtained from the FMCNA Knowledge Center data warehouse and were collected according to standard clinical protocols [8]. To standardize data reporting across cases and controls, an index date for each control was designated as the date of CPA in the matched case. Demographic data including age, sex, and race were determined as of January 1, 2010. Body mass index and comorbidity status including diabetes, coronary artery disease (CAD), and heart failure were determined as of the CPA event date in cases and at the corresponding index date in matched controls. The following dialytic session data were considered for the 30-day period immediately preceding, but not including, the index date: vascular access type, mean treatment time, mean interdialytic weight gain (IDWG), mean pre-HD heart rate, total number delivered treatments, and total number missed treatments. Biochemical data, erythropoietin stimulating agent (ESA) dose, and estimated dry weight were considered as the last value in the 30 days preceding index date. Dialysate composition was determined from the active physician order on the index date and was categorized based on typical clinical thresholds. Date of death and attributed cause of death were recorded by HD unit staff.

### 2.3. Statistical Analyses

Baseline subject characteristics were described across cases and controls as counts and proportions for categorical variables and as means and standard deviations for continuous variables. Two-group comparisons were made using Student's *t*-test of means or contingency table *χ* ^2^ test of proportions.

**Figure 1:**
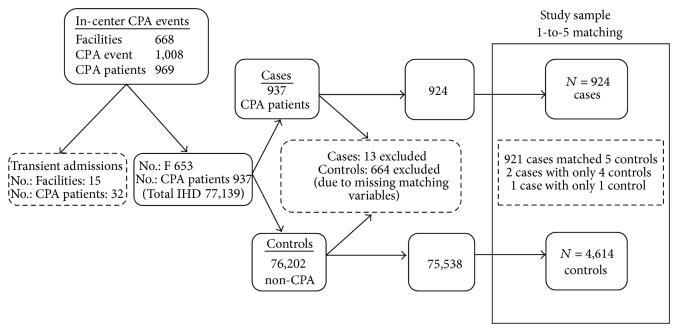
Random case-control matching without replacement for primary analysis. CPA, cardiopulmonary arrest; IHD, in-center hemodialysis.

For the primary analysis, each CPA case was randomly matched (without replacement) with up to 5 non-CPA controls on the basis of age (±3 years), sex, race (white/black/other), and diabetes status. Matching on factors strongly associated with mortality and/or sudden cardiac events was performed to minimize confounding from these previously established, nonmodifiable risk factors. Matching criteria data were available for 924 out of 937 (98.6%) cases and 75,538 out of 76,202 (99.1%) controls. Each of the 924 cases matched to 5 controls with the exception of 2 cases that matched to 4 controls and 1 case that matched to only 1 control; the primary cohort consisted of 4,614 controls (Figure 1).

Candidate CPA predictors were identified based on literature precedent [4, 6, 7, 9–11] and clinical relevance. Univariate logistic regression models (model 1) were used to identify potential CPA risk factors by estimating the CPA odds ratio (OR) for each of the prespecified variables. To identify variables that are independently associated with CPA, 3 additional multivariate logistic models were considered: model 2: multivariate model with inclusion of all covariates; model 3: multivariate model derived through stepwise regression with 0.05 as inclusion and elimination thresholds for significance; and model 4: from model 3 with the addition of quadratic terms for biochemical data to account for nonlinear associations. Model goodness-of-fit was evaluated with Hosmer-Lemeshow testing. The model c-statistic was used to estimate the model predictive capacity of each model. The relative contribution of each model covariate to CPA prediction was ranked according to the magnitude of the respective chi-square value in the final model.

**Figure 2:**
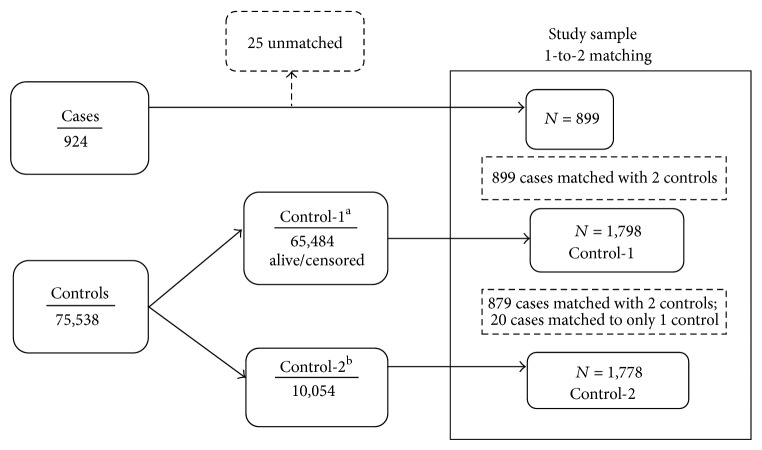
Random case-control matching without replacement for secondary analyses. ^a^Control-1 included those patients without peridialytic cardiopulmonary arrest who survived 2010. ^b^Control-2 included those patients without peridialytic cardiopulmonary arrest who died in 2010.

In secondary analyses, two pools of non-CPA matching controls were identified. Control-1s were defined as non-CPA patients who survived 2010, and control-2s were defined as non-CPA patients who died in 2010. Among the 75,538 eligible controls with adequate matching data, 65,484 (86.7%) met criteria for the control-1 pool, and 10,054 (13.3%) met criteria for the control-2 pool. Twenty-five out of 924 cases were unable to be matched to control-2s on the basis of age (±3 years), sex, race (white/black/other), and diabetes status; 899 cases were included in the secondary analysis. Each of the 899 cases was then similarly matched (random without replacement) to 2 patients from control-1, resulting in 1,798 control-1s for analysis. 879 cases matched with 2 control-2s. The remaining 20 cases matched with only 1 control-2 patient. Thus, a total of 1,778 control-2s were available for analysis (Figure 2). Among the control-1 patients, 169 were in the primary analysis control group while 282 control-2 patients were in the primary analysis control group. CPA cases were then compared to each of control-1s and to control-2s via logistic models constructed similar to the primary analysis.

Effect modification of the associations between CPA and individual prespecified covariates was explored through subgroup analyses. Sensitivity analyses with dialysate bicarbonate alone (versus total buffer) and comparative use of continuous versus categorical variables were performed.

With a large number of CPA events, our study is adequately powered to detect associations between the candidate associate factors and CPA. Assuming a baseline probability of CPA risk of 0.1, we have 90% power (*α* = 0.05) to detect an odds ratio of 1.16 across dichotomized independent variables. All analyses were implemented using SAS v9.3 (http://www.sas.com/).

## 3. Results

### 3.1. Baseline Characteristics

Table 1 displays the characteristics of the primary cohort cases and matched controls. For cases, the mean age was 65.7 years; 50.1% were male; 60.2% were white; 34.6% were black; 5.2% were of other racial ancestry; 71.5% were diabetic. Cases and matched controls were well balanced on these matching factors. Overall, the CPA and matched non-CPA controls were similar in terms of estimated dry weight, absolute IDWG, delivered treatment time, dialysate composition (sodium, potassium, calcium, and dialysate buffer concentrations), and the number of missed treatments due to unexcused absences. Compared to controls, CPA cases had shorter dialytic vintage; were more likely to dialyze via catheter; had more CAD and heart failure; had higher % IDWG; and received higher doses of ESA. CPA cases were also more likely to have lower serum albumin, hemoglobin, phosphorus, calcium, potassium, creatinine, normalized protein catabolic rate (nPCR), and eKt/V than controls. Controls were more likely to have lower serum bicarbonate. CPA cases were more likely to have been discharged from the hospital within 30 days prior to the CPA event.

### 3.2. Primary Analyses

**Figure 3:**
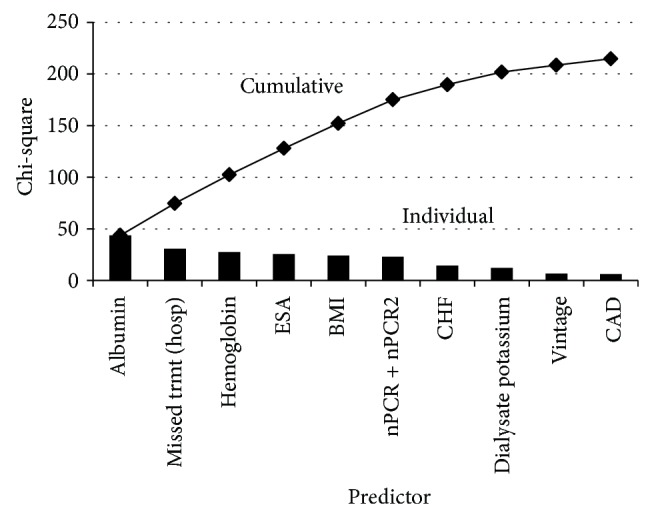
Relative contributions of predictors of peridialytic cardiopulmonary arrest in the primary analysis^a^. ^a^Based on model 4 chi-square values. nPCR, normalized protein catabolic rate; CAD, coronary artery disease; and ESA, erythropoietin stimulating agent. Note: missed treatment was due to hospitalization.

Table 2 displays the CPA model-building results. On univariate analysis (Model 1), CAD, heart failure, catheter access, lower body mass index, larger % IDWG, treatment time shortened by >10 minutes, missed treatments due to hospitalization, greater ESA dose, and higher serum bicarbonate were associated with increased odds of CPA. Longer vintage, higher dialysate calcium, eKt/V, albumin, hemoglobin, phosphorus, serum calcium, serum potassium, and nPCR were associated with decreased odds of CPA. After adjustment for confounders and accounting for nonlinear associations (Model 4), CAD, heart failure, missed HD treatments due to hospitalization, lower dialysate potassium, lower albumin, lower body mass index, lower hemoglobin, higher serum calcium, greater ESA dosing, and both low and high nPCR (J-shaped risk curve; quadratic) were associated with higher CPA odds. Hemoglobin, albumin, body mass index, ESA dosing, and missed treatment sessions due to hospitalization had the greatest predictive influence based on relative chi-square values (Figure 3). Dialysate sodium, calcium, and total buffer were not significantly associated with CPA. Considering dialysate bicarbonate in lieu of total buffer did not result in a significant association with CPA.

The data suggested effect modification of the CPA-serum calcium association on the basis of albumin. At lower albumin levels, higher serum calcium was associated with CPA; however, at higher albumin levels, higher serum calcium was not associated with CPA (data not shown). No effect modification between albumin and other model variables was detected. There was no effect modification of the CPA-serum calcium association by dialysate calcium and no effect modification of the CPA-dialysate potassium association by serum potassium (data not shown).

Timing of death with respect to the CPA event (index) date was compared across cases and controls (Table 3). A total of 299 (32.4%) of CPA cases died on the date of the CPA event compared to 9 (0.2%) of the controls; 548 (59.3%) cases died within one year of CPA compared to 1,333 (28.9%) of controls.

### 3.3. Secondary Analyses

To mitigate confounding by ambient health status, we matched CPA cases to non-CPA patients who survived 2010 (control-1) and, separately, to non-CPA patients who died in 2010 (control-2). Overall, control-2s were more similar to cases than control-1s (Table 4). Specifically, cases and control-2s were similar in terms of weight, CAD, heart failure, treatment time, shortening of treatment time, ESA dosing, number of missed treatments, eKt/V, albumin, phosphorus, calcium, bicarbonate, and nPCR. Control-1s had greater body weights, less CAD, heart failure, catheter use (compared to graft/fistula), missed treatments, and greater eKt/V, albumin, hemoglobin, phosphorus, calcium, potassium, creatinine, and nPCR compared to CPA cases.

Comparing cases to control-1s in the final model (model 4), lower body mass index, CAD, heart failure, longer vintage, catheter access, more missed treatments due to hospitalization, lower dialysate potassium, higher ESA dosing, and nPCR^2^ (quadratic) were associated with increased CPA odds; and higher BMI, albumin, hemoglobin, and nPCR were associated with decreased CPA odds (Table 5). Comparing cases to control-2s in the final model (model 4), higher serum calcium was associated with increased CPA odds, and higher hemoglobin, serum potassium, and ferritin were associated with decreased CPA odds (Table 5). Albumin, missed treatments due to hospitalization, ESA dose, hemoglobin, and nPCR had the greatest predictive influence on CPA based on relative chi-square values when comparing cases to control-1s; and serum potassium and hemoglobin had the greatest influence on CPA comparing cases to control-2s (see Supplementary Figures 1(A) and 1(B) in Supplementary Material available online at http://dx.doi.org/10.1155/2014/961978). Consistent with the primary analyses, dialysate total buffer and its bicarbonate component (separately) were not associated with CPA in either control-1 or control-2 secondary analyses.

## 4. Discussion

Peridialytic CPA is a rare event with a high subsequent fatality rate. In this case-control study inclusive of both cardiac and respiratory events, we demonstrated that comorbidities (CAD and heart failure), health status surrogates (lower body mass index, lower albumin, lower nPCR, lower hemoglobin, and recent hospitalizations), and procedural factors (lower dialysate potassium and higher ESA dosing) were associated with peridialytic CPA. The data suggest that lower hemoglobin, lower albumin, lower body mass index, higher ESA dosing, and greater preevent missed treatment sessions due to hospitalization most strongly associate with ambulatory HD facility CPA.

Multiple studies [4, 6, 10–16] have examined peridialytic SCA and SCD risk factors, but event definition inconsistencies have limited firm conclusions. In the cardiology literature, SCD is defined as the “unexpected natural death from a cardiac cause within a short time period, generally ≤1 hour from the onset of symptoms” [17]. Applying this definition to the general population, SCD occurs at a rate of 1 per 1,000 patient years [18]. The USRDS reports a SCD rate of 59 deaths per 1,000 HD patient years but defines SCD as death from arrhythmia or cardiac arrest of unknown cause [1]. In fact, 51.1 of the 59 deaths per 1,000 years are reported as “cardiac arrest cause unknown,” suggesting that the SCD estimate may be inflated in the USRDS data [1, 18]. SCD risk factor investigations have relied on different outcome definitions as exemplified by Genovesi et al.'s definition of sudden death as an unexpected natural death within 1 hour of the onset of symptoms [10] versus Parekh et al.'s definition of SCD as an out-of-hospital death with an underlying cardiac cause [13]. The SCA literature suffers from similar definition discrepancies [4, 6, 10, 16]. Differing outcome specification prevents meta-analysis and renders the study of an already rare event even more challenging. In this analysis, we examined associates of CPA, defined as the absence of a pulse or cessation of breathing during HD or immediately preceding or following the HD procedure while the patient is in the dialysis facility. This definition allowed us to investigate a broad class of events associated with not only morbidity and mortality but also significant emotional stress to dialysis facility staff and fellow patients. Better understanding of CPA associates as examined in our study may not only improve patient outcomes but also improve the patient facility experience.

Regardless of the outcome investigated, the peridialytic period is incontrovertibly a high risk period. Interdialytic volume and metabolite accumulation followed by obligate intradialytic electrolyte and fluid shifts may promote a proarrhythmic environment. Numerous studies have demonstrated increased arrhythmias in the peridialytic period [19–22] and have linked serum and dialysate electrolyte profiles to QT interval abnormalities [23–28]. Conduction abnormalities may be induced by gradient driven electrolyte shifts, transient episodes of hypokalemia and/or hypocalcemia, and/or HD procedure-related myocardial ischemia. Our findings of an association between CPA and lower dialysate potassium support those of other studies [4, 6, 11, 12] and suggest that electrolyte-mediated conduction abnormalities may play important roles in peridialytic CPA.

We hypothesized that other dialysate/serum components such as calcium and bicarbonate might associate with CPA. Consistent with Pun et al., we found an association between higher serum calcium and CPA; however, we found no CPA association with dialysate calcium or the serum-dialysate calcium gradient as did Pun et al. [7]. Such discrepancy may have resulted from our outcome of CPA (versus SCD in Pun et al.'s analysis) [7]. Serum and dialysate bicarbonate levels are also plausible SCD associates as higher dialysate bicarbonate has been linked to increased mortality [29] and SCA [6]. Alkalosis-driven electrolyte shifts could underlie this association; however, we found no association between CPA and serum bicarbonate or dialysate total buffer (bicarbonate + acetate), particularly with adjusted models that included nutritional markers. Furthermore, sensitivity analyses examining dialysate total buffer and dialysate bicarbonate in the models (separately) demonstrated no association between either and CPA risk.

Markers of poor health and chronic inflammation including lower body mass index, lower albumin, lower nPCR, greater ESA dosing, lower hemoglobin, and greater missed treatment due to hospitalizations also associated with CPA in our cohort. These findings should not be surprising given the association of malnutrition and inflammation with poor outcomes among HD patients [30–34] and their association with atherosclerotic cardiovascular disease [35–37]. It is not known if greater inflammation and malnutrition trigger atherosclerosis progression or if they reflect underlying cardiovascular disease burden. Similarly, our finding of an association between CPA risk and lower hemoglobin and higher ESA dosing may stem from ESA resistance related to underlying inflammation, nutrient deficiencies, malignancy, or other systemic illness [38]; all such conditions portend poor outcomes.

To address confounding by ambient health status, we performed secondary analyses in which we matched CPA cases with non-CPA controls who survived 2010 (control-1s) and, separately, with non-CPA controls who died in 2010 (control-2s). Not surprisingly, control-2s were more similar to cases than were control-1s. Comparing CPA cases to patients who died in 2010, we demonstrated that lower hemoglobin, higher serum calcium, lower serum potassium, and lower ferritin are associated with increased odds of peridialytic CPA. These findings suggest that these characteristics play roles beyond that of health status surrogates in their CPA associations.

It must be emphasized that ambulatory HD facility CPA occurrence is dwarfed by community CPA events. Therefore, our study should not detract from the importance of global CPA prevention. However, targeting in-facility CPA represents a unique prevention opportunity given the feasibility of HD prescription modification and the accessibility of immediate medical intervention. Despite being a relatively rare event, SCA is an important cause of mortality among HD patients. In our cohort, 32.4% of patients who experience CPA died on the CPA event date, and an additional 16.8% died within 30 days. Thus, the identification of high risk subgroups will inform preventive dialytic treatment strategies. HD modifications such as avoidance of low potassium baths (<2 mEq/L) and use of the lowest effective ESA dosing may be important in reducing CPA. Additionally, attention should be paid to nutritional status, underlying illness, and inflammatory processes as these conditions may render HD patients susceptible to peridialytic CPA.

The strengths of our study include its large, nationally representative cohort with a large number of CPA events, the breadth of available covariates, and case-control matched study design. Several limitations of our study deserve mention. As with all observational studies, our study may contain bias due to imperfect control for confounding. We attempted to limit this confounding by matching subjects on age, gender, race, and diabetic status. Such matching accounts for confounding not only from these variables but also from variables that are strongly associated with these variables. Because of data limitations, we were unable to account for additional cardiovascular status markers such as cardiac troponin, cardiac structural abnormalities, residual renal function, or additional inflammatory markers such as CRP and IL-6. We cannot exclude the possibility of residual confounding related to these variables or other unconsidered variables. In our primary analysis, we allowed CPA cases to match to patients who died later in the study year in efforts to best represent the CPA risk across all patients. It is possible that residual confounding from ambient health status may have influenced results in the primary analysis. Second, we specified CPA as the outcome in all analyses. 67.6% of CPA cases survived beyond the day of CPA, suggesting that the etiology of their CPA may have been respiratory in nature rather than cardiac. Our results should not be extrapolated to other outcomes such as SCA or SCD. On a related note, we lacked data on the breakdown of respiratory versus cardiac CPA events and thus cannot perform event-specific risk factor analyses. Finally, we lacked data regarding the timing of CPA relative to the dialysis treatment and are thus unable to determine whether the identified risk factor-CPA associations differ across the pre-, intra-, and postdialytic time periods.

## 5. Conclusions

This study demonstrates that, among chronic outpatient HD patients, CAD, heart failure, missed treatments due to hospitalization, lower dialysate potassium, higher ESA dosing, lower albumin, lower body mass index, lower hemoglobin, lower (and much higher) nPCR, and higher serum calcium are associated with increased odds of ambulatory HD facility CPA. Of these, missed treatments due to hospitalization, albumin, hemoglobin, body mass index, and ESA dosing had the greatest influence on CPA. Further prospective studies are needed to confirm and generalize findings and to explore interventional strategies aimed at mitigating CPA risk among chronic HD patients.

## Supplementary Material

The supplemental material contains graphical representations of the relative contributions of predictors of peridialytic CPA in the secondary analysis with control-1s (Supplemental Figure 1(A)) and with control-2s (Supplemental Figure 1(B)).

## Figures and Tables

**Table 1:** Characteristics of the primary study cohort across cases and matched controls^a^.

Variable	CPA patients (cases)	Non-CPA patients (controls)
	n = 924	*n* = 4,614
Matching factors		
Age (years)		
Mean ± SD	65.7 ± 13.2	65.7 ± 13.1
Median [IQR]	66.3 [58.2, 75.5]	66.1 [58.0, 75.3]
(Min, max)	(24.7, 96.2)	(23.3, 95.5)
Male	463 (50.1%)	2309 (50.0%)
Race		
White	556 (60.2%)	2780 (60.3%)
Black	320 (34.6%)	1595 (34.6%)
Other	48 (5.2%)	239 (5.2%)
Diabetes	661 (71.5)	3304 (71.6%)

Other variables		
Weight (kg)	77.5 ± 23.1	79.0 ± 22.0
Body mass index (kg/m²)		
<18.5	51 (5.8%)	141 (3.1%)
18.6–25	228 (25.9%)	1301 (28.9%)
25.1–29.9	318 (36.1%)	1750 (38.9%)
≥30	284 (32.2%)	1306 (29.0%)
Coronary artery disease	271 (29.3%)	1136 (24.6%)^**^
Heart failure	326 (35.3%)	1403 (30.4%)^**^
Vintage^b^ (days)	1,088 ± 1,114	1,208 ± 1,205^**^
<120 days	207 (22.4%)	483 (10.5%)
20 days–<1 year	117 (12.7%)	715 (15.5%)
1 year–<3 years	215 (23.3%)	1440 (31.2%)
3 years–<5 years	165 (17.9%)	862 (18.7%)
5 years or more	193 (20.9%)	1029 (22.3%)
Missing	27 (2.9%)	85 (1.8%)
Access type^c^		
Catheter	416 (46.2%)	1560 (34.8%)^****^
Fistula	319 (35.4%)	2010 (44.8%)
Graft	166 (18.4%)	919 (20.5%)
IDWG (kg)	3.0 ± 8.7	2.6 ± 4.9
Percent IDWG (%)	4.2 ± 10.5	3.5 ± 5.1^**^
Patients discharged from hospital within 30 days prior to CPA	54 (5.8%)	182 (3.9%)^**^
Treatment time^d^ (minutes)		
Prescribed time	226 ± 34	223 ± 37^*^
Delivered time	218 ± 46	219 ± 44
Patients with treatment time shortened > 10 minutes	179 (20.2%)	724 (16.1%)^**^
Dialysate sodium (mEq/L)	137.8 ± 2.5	137.8 ± 2.5
	*n* = 745	*n* = 3,774
Dialysate calcium (mEq/L)		
<2.3	214 (28.7%)	1057 (28.0%)
2.3–2.5	513 (68.9%)	2566 (68.0%)
>2.5	18 (2.4%)	151 (4.0%)
	*n* = 745	*n* = 3,774
Dialysate potassium (mEq/L)		
1	31 (4.2%)	126 (3.3%)
2	564 (75.7%)	2883 (76.4%)
>2	150 (20.1%)	765 (20.3%)
	*n* = 745	*n* = 3,774
Dialysate buffer^e^ (mEq/L)		
<41	92 (12.4%)	455 (12.1%)
41–45	602 (80.8%)	2999 (79.5%)
>45	51 (6.9%)	320 (8.5%)
	*n* = 745	*n* = 3,774
Dialysate bicarbonate (mEq/L)	36.5 ± 1.9	36.4 ± 2.3
	*n* = 745	*n* = 3,774
Dialysate acetate (mEq/L)	6.5 ± 2.0	6.6 ± 2.0
	*n* = 745	*n* = 3,774
ESA dose per treatment (units)	11,233 ± 8,656	8,562 ± 7,994^****^
Number of treatments	10.0 ± 3.6	11.4 ± 2.7^****^
Number of missed treatments	1.47 ± 2.78	0.87 ± 2.08^****^
Number of missed treatments due to hospitalization	1.21 ± 2.69	0.63 ± 1.91^****^
Number of missed treatments due to unexcused absence	0.26 ± 0.87	0.24 ± 0.84
eKt/V	1.45 ± 0.45	1.49 ± 0.40^**^
Albumin (g/dL)	3.5 ± 0.5	3.7 ± 0.5^****^
Hemoglobin (g/dL)	10.8 ± 1.5	11.4 ± 1.3^****^
Phosphorus (mg/dL)	5.0 ± 1.7	5.2 ± 1.7^***^
Calcium (mg/dL)	8.9 ± 0.8	9.0 ± 0.8^***^
Potassium (mEq/L)	4.6 ± 0.8	4.7 ± 0.7^****^
Ferritin (ng/mL)	662 ± 531	676 ± 611
Bicarbonate (mEq/L)	24.1 ± 3.8	23.8 ± 3.5^*^
Creatinine (mg/dL)	6.5 ± 2.6	7.3 ± 2.8^****^
nPCR (g/kg/day)	0.86 ± 0.31	0.93 ± 0.29^****^

^a∗^ *P* < 0.05; ^**^ *P* < 0.01; ^***^ *P* < 0.001; ^****^ *P* < 0.0001; based on two-sample Student's *t*-testing or chi-square testing as dictated by data type. Values reported as means ± SD or *n* (%) as dictated by data type.

^b^Vintage defined as number of days from the date of first ever dialysis to the date of event (CPA). For controls, event date was assigned the same index date from the matched CPA case.

^c^Catheter compared to fistula/graft.

^d^Mean treatment time in the 30 days prior to CPA (excludes CPA treatment).

^e^Dialysate buffer = dialysate bicarbonate + dialysate acetate.

**Table 2:** Logistic models of peridialytic cardiopulmonary arrest predictors in the matched primary analysis.

	Model 1	Model 2	Model 3	Model 4
	(univariate)	(full multivariate)	(stepwise)	(Model 3 + quadratics)
	OR (95% CI)	OR (95% CI)	OR (95% CI)	OR (95% CI)
Body mass index (kg/m²)				
<18.5	1.66^*^ (1.05–2.65)	1.95^*^ (1.10–3.44)	2.03^***^ (1.37–3.00)	1.95^***^ (1.32–2.89)
18.6–25	1 (ref.)	1 (ref.)	—	—
25.1–29.9	0.81 (0.62–1.04)	0.85 (0.61–1.19)	—	—
≥30	0.84 (0.66–1.06)	0.80 (0.52–1.23)	—	—
Coronary artery disease	1.27^**^ (1.09–1.49)	1.41^***^ (1.15–1.72)	1.39^***^ (1.14–1.70)	1.38^**^ (1.13–1.68)
Heart failure	1.25^**^ (1.08–1.45)	1.24^*^ (1.02–1.51)	1.24^*^ (1.03–1.51)	1.27^*^ (1.05–1.54)
Vintage (per 100 days)	0.99^*^ (0.98–1.00)	1.00 (0.99–1.01)	—	—
Catheter vascular access	1.61^****^ (1.39–1.86)	1.00 (0.82–1.22)	—	—
EDW (kg)	1.00 (0.99–1.00)	1.00 (1.00–1.01)	—	—
Percent IDWG (%)	1.01^**^ (1.004–1.02)	1.00 (0.96–1.04)	—	—
TT shortened > 10 minutes (yes/no)	1.32^**^ (1.10–1.58)	1.14 (0.89–1.45)	—	—
Missed treatment due to hospitalization (yes/no)	1.94^****^ (1.65–2.28)	1.48^***^ (1.20–1.84)	1.48^***^ (1.20–1.82)	1.45^***^ (1.18–1.80)
Missed treatment due to unexcused absence (yes/no)	1.05 (0.85–1.28)	1.01 (0.77–1.33)	—	—
Dialysate sodium (mEq/L)	0.99 (0.96–1.02)	1.01 (0.97–1.04)	—	—
Dialysate calcium (mEq/L)			—	—
<2.3	1.01 (0.82–1.25)	1.07 (0.83–1.37)	—	—
2.3–2.5	1.00 (ref.)	1.00 (ref.)	—	—
>2.5	0.60 (0.33–1.10)	0.64 (0.32–1.30)	—	—
Dialysate potassium (mEq/L)				
1	1.26 (0.77–2.06)	1.72^*^ (0.99–2.99)	1.73^**^ (1.12–2.68)	1.89^**^ (1.23–2.90)
2	1.00 (ref.)	1.00 (ref.)	—	—
>2	1.00 (0.79–1.28)	0.83 (0.61–1.12)	—	—
Dialysate buffer (mEq/L)			—	—
<41	1.01 (0.75–1.35)	0.95 (0.67–1.34)	—	—
41–45	1.00 (ref.)	1.00 (ref.)	—	—
>45	0.79 (0.55–1.16)	0.77 (0.49–1.19)	—	—
ESA per treatment (per 1000 u)	1.04^****^ (1.03–1.05)	1.01^*^ (1.00–1.02)	1.01^*^ (1.00–1.03)	1.01^*^ (1.00–1.02)
eKt/V	0.73^**^ (0.59–0.91)	0.93 (0.72–1.22)	—	—
Albumin (g/dL)	0.43^****^ (0.38–0.50)	0.65^***^ (0.51–0.81)	0.65^****^ (0.53–0.81)	0.68^***^ (0.55–0.85)
Hemoglobin (g/dL)	0.73^****^ (0.69–0.77)	0.81^****^ (0.76–0.88)	0.82^****^ (0.76–0.88)	0.81^****^ (0.75–0.87)
Phosphorus (mg/dL)	0.93^***^ (0.89–0.97)	1.01 (0.95–1.07)	—	—
Calcium (mg/dL)	0.84^***^ (0.77–0.92)	1.17^*^ (1.02–1.33)	1.16^*^ (1.02–1.32)	1.17^*^ (1.04–1.33)
Potassium (mEq/L)	0.78^****^ (0.71–0.86)	0.85^*^ (0.74–0.98)	—	—
Ferritin (per 100 ng/mL)	1.00 (0.98–1.01)	0.99 (0.97–1.01)	—	—
Bicarbonate (mEq/L)	1.03^*^ (1.01–1.05)	0.99 (0.96–1.01)	—	—
nPCR (g/kg/day)	0.45^****^ (0.34–0.60)	0.69 (0.47–1.00)	0.62^**^ (0.45–0.88)	0.53^***^ (0.38–0.75)
nPCR^2^(g/kg/day)	—	—	—	1.99^**^ (1.24–3.20)

c-statistic	—	0.68	0.67	0.68

^*^ *P* < 0.05; ^**^ *P* < 0.01; ^***^ *P* < 0.001; ^****^ *P* < 0.0001.

For independent variables with more than 2 categories, *P* values and confidence intervals were adjusted with multiple comparisons using Bonferroni method.

OR, odds ratio; CI, confidence interval; IDWG, interdialytic weight gain; TT, treatment time; nPCR, normalized protein catabolic rate; ESA, erythropoietin stimulating agent; and EDW, estimated dry weight.

**Table 3:** Death following peridialytic cardiopulmonary arrest events across cases and controls.

	CPA patients *N* (%)	Controls^a^ *N* (%)
Death on date of CPA	299 (32.4)	9 (0.2)^****^
Death within 1–30 days following CPA	155 (16.8)	144 (3.1)^****^
Death between 31 and 365 days from CPA	94 (10.2)	1,180 (25.6)^****^

Total deaths at 1 year	548 (59.3)	1,333 (28.9)^****^
Total surviving at 1 year or censored by reasons other than death	376 (40.7)	3,281 (71.1)^****^

Total	924	4,614

^a^For control patients, the reference date is the CPA date for the matched CPA case.

^****^ *P* < 0.0001.

**Table 4:** Characteristics of the secondary study cohort across cases and matched control-1s and control-2s^a^.

Variable	Patients with CPA in 2010	Patients without CPA who	
		survived 2010	died in 2010
	(cases)	(control-1s)	(controls-2s)
	*N* = 899	*N* = 1,798	*N* = 1,778
Matching factors			
Age (years)			
Mean ± SD	66.1 ± 12.7	66.0 ± 12.5	66.2 ± 12.5
Median [IQR]	66.4 [58.5, 75.5]	66.2 [58.5, 75.4]	66.4 [58.5, 75.5]
(Min, max)	(27.4, 96.2)	(24.5, 94.1)	(24.5, 94.6)
Male	456 (50.7%)	912 (50.7%)	901 (50.7%)
Race			
White	550 (61.2%)	1100 (61.2%)	1095 (61.6%)
Black	306 (34.0%)	612 (34.0%)	602 (33.9%)
Other	43 (4.8%)	86 (4.8%)	81 (4.6%)
Diabetes	649 (72.2%)	1298 (72.2%)	1284 (72.2%)

Other variables			
Weight (kg)	78.8 ± 23.0	81.2 ± 22.4^***^	76.8 ± 23.0
Body mass index (kg/m²)			
<18.5	47 (5.4%)	32 (1.8%)^****^	95 (5.5%)
18.6–25	240 (27.4%)	554 (31.4%)	461 (26.6%)
25.1–29.9	315 (36%)	763 (43.2%)	607 (35.0%)
≥30	273 (31.2%)	416 (23.6%)	570 (32.9%)
Coronary artery disease	268 (29.8%)	422 (23.5%)^***^	495 (27.8%)
Heart failure	317 (35.3%)	464 (25.8%)^****^	623 (35.0%)
Vintage^b^ (days)	1097 ± 1121	1136 ± 1136	1249 ± 1299^**^
Access type^c^			
Catheter	404 (46.1%)	513 (29.5%)^****^	40.7%^*^
Fistula	312 (35.6%)	872 (50.1%)	39.00%
Graft	161 (18.4%)	356 (20.5%)	20.30%
IDWG (kg)	2.5 ± 1.3	2.5 ± 1.1	2.5 ± 1.3
Percent IDWG (%)	3.2 ± 1.6	3.2 ± 1.3	3.3 ± 1.6^*^
Patients discharged from hospital within 30 days prior to CPA	54 (6.0%)	47 (2.6%)^****^	115 (6.5%)
Treatment time^d^ (minutes)			
Prescribed time	226 ± 34	222 ± 37^**^	224 ± 38
Delivered time	218 ± 44	219 ± 43	217 ± 44
Time shortened	12 ± 32	7 ± 24^****^	11 ± 30
Patients with treatment time shortened > 10 minutes	174 (20.2%)	242 (13.8%)^****^	338 (19.6%)
Dialysate sodium (mEq/L)	137.8 ± 2.5	137.8 ± 2.2	137.9 ± 2.9
	*n* = 725	*n* = 1,477	*n* = 1,480
Dialysate calcium (mEq/L)			
<2.3	206 (28.4%)	408 (27.6%)	396 (26.8%)
2.3–2.5	501 (69.1%)	1022 (69.2%)	1015 (68.6%)
>2.5	18 (2.5%)	47 (3.2%)	69 (4.7%)
	*n* = 725	*n* = 1,477	*n* = 1,480
Dialysate potassium (mEq/L)			
1	29 (4.0%)	38 (2.6%)	49 (3.3%)
2	550 (75.9%)	1148 (77.7%)	1103 (74.5%)
>2	146 (20.1%)	291 (19.7%)	328 (22.2%)
	*n* = 725	*n* = 1,477	*n* = 1,480
Dialysate buffer^e^(mEq/L)			
<41	88 (12.1%)	174 (11.8%)	198 (13.4%)
41–45	587 (81.0%)	1204 (81.5%)	1174 (79.3%)
>45	50 (6.9%)	99 (6.7%)	108 (7.3%)
	*n* = 725	*n* = 1,477	*n* = 1,480
ESA dose per treatment (units)	9,313 ± 7,713	6,033 ± 6,374^****^	9,934 ± 8,630
Number of treatments	10.6 ± 3.9	12.3 ± 2.2^****^	11.0 ± 3.5^**^
Number of missed treatments	1.5 ± 2.8	0.56 ± 1.5^****^	1.5 ± 2.9
Number of missed treatments due to hospitalization	1.2 ± 2.7	0.37 ± 1.4^****^	1.1 ± 2.8
Number of missed treatments due to unexcused absence	0.25 ± 0.86	0.19 ± 0.65^*^	0.34 ± 0.99^*^
eKt/V	1.44 ± 0.3	1.49 ± 0.3^**^	1.46 ± 0.8
Albumin (g/dL)	3.5 ± 0.6	3.8 ± 0.4^****^	3.5 ± 0.5
Hemoglobin (g/dL)	10.8 ± 1.5	11.5 ± 1.2^****^	11.1 ± 1.5^****^
Phosphorus (mg/dL)	5.0 ± 1.7	5.2 ± 1.6^**^	5.15 ± 1.8
Calcium (mg/dL)	8.9 ± 0.8	9.0 ± 0.7^****^	8.8 ± 0.8
Potassium (mEq/L)	4.6 ± 0.8	4.8 ± 0.7^****^	4.7 ± 0.8^***^
Ferritin (ng/mL)	660 ± 533	662 ± 423	729 ± 803^*^
Bicarbonate (mEq/L)	24.1 ± 3.8	23.8 ± 3.4^*^	23.9 ± 3.6
Creatinine (mg/dL)	6.5 ± 2.6	7.7 ± 2.8^****^	6.8 ± 2.6^**^
nPCR (g/kg/day)	0.86 ± 0.30	0.96 ± 0.28^****^	0.89 ± 0.52

^a∗^ *P* < 0.05; ^**^ *P* < 0.01; ^***^ *P* < 0.001; ^****^ *P* < 0.0001; based on two-sample Student's *t*-testing or chi-square testing as dictated by data type. Values reported as means ± SD or *n* (%) as dictated by data type.

^b^Vintage defined as number of days from the date of first ever dialysis to the date of event (CPA). For controls, event date was assigned the same index date from the matched CPA case.

^c^Catheter compared to fistula/graft.

^d^Mean treatment time in the 30 days prior to CPA (excludes CPA treatment).

^e^Dialysate buffer = dialysate bicarbonate + dialysate acetate.

**Table 5:** Logistic models of predictors of peridialytic cardiopulmonary arrest in two matched secondary analyses.

Variable	CPA cases versus control-1s		CPA cases versus control-2s	
	Model 1	Model 4	Model 1	Model 4
	(univariate)	(stepwise + quadratics)	(univariate)	( stepwise + quadratics)
	OR (95% CI)	OR (95% CI)	OR (95% CI)	OR (95% CI)
Body mass index (kg/m^2^)				
<18.5	2.24^**^ (1.18–4.24)	3.52^****^ (1.98–6.26)	1.03 (0.62–1.72)	—
18.6–25	1 (ref.)	—	—	—
25.1–29.9	0.66^***^ (0.49–0.88)	—	1.09 (0.82–1.45)	—
≥30	0.63^****^ (0.48–0.83)	0.75^*^ (0.60–0.95)	1.08 (0.83–1.42)	—
Coronary artery disease	1.39^***^ (1.16–1.67)	1.36^*^ (1.07–1.73)	1.11 (0.93–1.33)	—
Heart failure	1.56^****^ (1.31–1.85)	1.57^****^ (1.25–1.99)	1.00 (0.85–1.19)	—
Vintage (per 100 days)	1.00 (1.00-1.00)	1.01^**^ (1.00–1.02)	1.00^*^ (1.00-1.00)	—
Catheter vascular access	2.05^****^ (1.73–2.42)	—	1.24^*^ (1.06–1.47)	—
Percent IDWG	0.99 (0.94–1.06)	—	0.94 (0.89–0.99)^*^	—
TT shortened > 10 minutes (yes/no)	1.11 (0.82, 1.51)	—	1.03 (0.80–1.33)	—
Missed treatment due to hospitalization (yes/no)	2.02^****^(1.54, 2.65)	2.13^****^ (1.63, 2.79)	1.25 (0.99, 1.56)	—
Missed treatment due to unexcused absence (yes/no)	1.08 (0.78, 1.50)	—	0.82 (0.62, 1.09)	—
Dialysate sodium (mEq/L)	1.00 (0.96–1.04)	—	0.99 (0.95–1.02)	—
Dialysate calcium (mEq/L)		—		
<2.3	1.03 (0.81–1.31)	—	1.05 (0.83–1.35)	—
2.3–2.5	1.00 (ref.)	—	1.00 (ref.)	—
>2.5	0.78 (0.40–1.54)	—	0.53 (0.28–1.01)	—
Dialysate potassium (mEq/L)				
1	1.59 (0.87–2.91)	2.67^***^ (1.54–4.63)	1.19 (0.67–2.11)	—
2	1.00 (ref.)	—	1.00 (ref.)	—
>2	1.05 (0.80–1.38)	—	0.89 (0.68–1.17)	—
Dialysate buffer (mEq/L)		—		
<41	1.04 (0.74–1.45)	—	0.89 (0.64–1.24)	—
41–45	1.00 (ref.)	—	1.00 (ref.)	—
>45	1.04 (0.67–1.60)	—	0.93 (0.60–1.42)	—
ESA per treatment (per 1000 u)	1.007^****^ (1.006–1.008)	1.04^****^ (1.02–1.06)	1.00 (1.00-1.00)	—
eKt/V	0.64^**^ (0.49–0.84)	—	0.95 (0.80–1.12)	—
Albumin (g/dL)	0.21^****^ (0.17–0.25)	0.40^****^ (0.30–0.52)	1.01 (0.87–1.17)	—
Hemoglobin (g/dL)	0.67^****^ (0.63–0.72)	0.78^****^ (0.72–0.86)	0.87^****^ (0.83–0.92)	0.87^****^ (0.82–0.92)
Phosphorus (mg/dL)	0.92^**^ (0.88–0.97)	—	0.96 (0.91–1.00)	—
Calcium (mg/dL)	0.72^****^ (0.65–0.80)	—	1.04 (0.95–1.15)	1.13^*^ (1.02–1.25)
Potassium (mEq/L)	0.75^****^ (0.67–0.85)	—	0.82^***^ (0.74–0.92)	0.84^**^ (0.75–0.93)
Ferritin (per 100 ng/ml)	1.00 (0.98–1.02)	—	0.98^*^ (0.97–1.00)	0.98^*^ (0.97–1.00)
Bicarbonate (mEq/L)	1.00 (1.00–1.05)	—	1.02 (0.99–1.04)	—
nPCR (g/kg/day)	0.25^****^ (0.18–0.34)	0.43^****^ (0.28–0.64)	0.79 (0.59–1.04)	—
nPCR^2^ (g/kg/day)	—	2.22^*^ (1.18–4.19)	—	—

c-statistic	—	0.77	—	0.58

^*^ *P* < 0.05; ^**^ *P* < 0.01; ^***^ *P* < 0.001; ^****^ *P* < 0.0001.

For independent variables with more than 2 categories, *P* values and confidence intervals were adjusted with multiple comparisons using Bonferroni method.

OR, odds ratio; CI, confidence interval; IDWG, interdialytic weight gain; TT, treatment time; nPCR, normalized protein catabolic rate; ESA, erythropoietin stimulating agent; and EDW, estimated dry weight.
