# Supplementary material for: Associates of Cardiopulmonary Arrest in the Perihemodialytic Period
Source: Int J Nephrol. 2014 Nov 4;2014:961978. doi: 10.1155/2014/961978 (PMC4235586; doi:10.1155/2014/961978)
Supplement: Supplementary file 1 — The supplemental material contains graphical representations of the relative contributions of predictors of peridialytic CPA in the secondary analysis with control-1s (Supplemental Figure 1(A)) and with control-2s (Supplemental Figure 1(B)). [file 961978.f1.docx]

**Supplemental Figure 1A.** Relative contributions of predictors of peri-dialytic cardiopulmonary arrest in the secondary analysis with control-1s

**Supplemental Figure 1B.** Relative contributions of predictors of peri-dialytic cardiopulmonary arrest in the secondary analysis with control-2s.
